# Protective Effect of EDC/NHS Cross-Linking Against Urea-Induced Collagen Destabilization in Ready-to-Eat Sea Cucumber During Room-Temperature Storage

**DOI:** 10.3390/foods15122117

**Published:** 2026-06-12

**Authors:** Jiarun Gao, Le Yu, Xiang Wan, Leilei Sun, Wenkui Song

**Affiliations:** 1 Guangdong Provincial Key Laboratory of Aquatic Products Processing and Safety, Guangdong Province Engineering Laboratory for Marine Biological Products, Guangdong Provincial Engineering Technology Research Center of Seafood, Key Laboratory of Advanced Processing of Aquatic Products of Guangdong Higher Education Institution, College of Food Science and Technology, Guangdong Ocean University, Zhanjiang 524088, China; 15863805335@163.com; 2 College of Life Science, Yantai University, Yantai 264005, China; ue040108@163.com (L.Y.); 15263023723@163.com (X.W.)

**Keywords:** ready-to-eat sea cucumber, *N*-(3-Dimethylaminopropyl)-N′-ethylcarbodiimide hydrochloride/*N*-hydroxysuccinimide, cross-linking treatment, hydrogen bonds, molecular docking

## Abstract

Ready-to-eat sea cucumbers (RSC) cannot be preserved at room temperature due to autolysis, which is closely related to the instability of collagen resulting from the disruption of hydrogen bonds. To investigate the protective effect of *N*-(3-Dimethylaminopropyl)-N′-ethylcarbodiimide hydrochloride/*N*-hydroxysuccinimide (EDC/NHS) cross-linking against disruption of hydrogen bonds and its role in stabilizing RSC quality at room temperature, this study designed comparative experiments involving EDC/NHS cross-linking treatments with varying sequences of hydrogen bonds disruption. The results indicated that EDC/NHS positively affects the stabilization of the collagen structure in RSC. The various quality parameters of both groups of RSC that underwent cross-linking treatment before and after hydrogen bonds disruption were significantly better than those of the control group, which only experienced the breaking of hydrogen bonds. Notably, the Eb group, which underwent EDC/NHS cross-linking treatment prior to the disruption of the hydrogen bonds network, yielded even more favorable results. Preliminary analyses of textural properties and moisture content suggested that EDC/NHS helps delay the deterioration of RSC quality. The levels of soluble components and carbonyl groups indicated that prior cross-linking treatment is more effective in mitigating collagen degradation and oxidation. Differential scanning calorimetry revealed that the reduction in ΔH for the Eb group was only 2.4%. Furthermore, fluorescence spectroscopy, Fourier transform infrared spectroscopy, and circular dichroism spectroscopy, examined from the perspectives of secondary and tertiary structures respectively, indicated that the cross-linking mechanism of EDC/NHS involves the formation of a more robust network of amide bonds, thereby preventing the disruption of hydrogen bonds and enhancing collagen stability, enabling it to better resist the cleavage of hydrogen bonds due to urea. The scanning electron microscope and Van Gieson’s staining techniques offer a clearer illustration of this point from a microscopic perspective. Moreover, molecular docking simulations have indicated the cross-linking mechanism of EDC/NHS at the atomic level, thereby establishing a scientific foundation for the potential application and development of EDC/NHS in room-temperature storage technologies for RSC.

## 1. Introduction

Sea cucumbers are marine invertebrates widely distributed in deep-sea regions globally, inhabiting coral reefs, mudflats, and seaweed habitats. They belong to the class Holothuroidea within the phylum Echinodermata [1]. Sea cucumbers contain various nutritional components that impart a range of biological and pharmacological effects, including antioxidant, antibacterial, and immunomodulatory activities [2]. Consequently, they are increasingly regarded as valuable sources for both food and medicinal applications [3]. However, fresh sea cucumbers are highly perishable and susceptible to degradation due to endogenous enzymes, microbial contamination, and environmental conditions, complicating their preservation and transport [4]. To address these challenges, traditional dehydration techniques, such as salting and air-drying, are commonly employed in processing [5]. Nevertheless, these methods often result in a considerable loss of water-soluble nutrients and flavor compounds, as the final product typically requires repeated washing and boiling prior to consumption. Ready-to-eat sea cucumbers (RSC), produced rapidly through low-temperature cooking and high-pressure sterilization, are highly favored by consumers for their convenience, flavor retention, and nutritional value [6]. However, studies indicate that RSC also experiences body wall degradation during storage, significantly impacting product quality [7]. Chen et al. [8] investigated the self-degradation of RSC at 4 °C, revealing that collagen constitutes the primary component of RSC body walls. Prolonged storage significantly degrades the quality of RSC, leading to the degradation of collagen fibers. Additionally, Peng et al. [9] described the deterioration of RSC quality as a result of non-enzymatic self-degradation. The stability of the spatial structure of sea cucumber collagen is maintained by a network of intra- and intermolecular chemical bonds, predominantly hydrogen bonds and hydrophobic interactions. Zhang et al. [10] demonstrated the critical role of hydrogen bonds, which are formed by the hydroxyl (-OH) of collagen, in stabilizing the collagen triple helix. Their study revealed that the disruption of hydrogen bonds in RSC due to urea exposure led to the progressive degradation of collagen fibers, with the degree of degradation directly correlating with urea concentration. This finding confirms that hydrogen bonds are among the primary contributors to the structural stability of collagen.

Previous studies have explored cross-linking strategies aimed at enhancing the resistance of the collagen network to degradation. Zhu et al. [11] demonstrated that treatment with celery extract or chlorogenic acid significantly improved the quality of RSC. Additionally, Yu et al. [12] reported that a polyphenol extract derived from Fucus vesiculosus could inhibit oxidative reactions by scavenging free radicals, thereby delaying the degradation of collagen in the sea cucumber body wall during heat treatment. The antioxidant activity of this extract was confirmed as the primary protective mechanism. Although these natural polyphenol-based cross-linking strategies have demonstrated some success in improving the stability of RSC collagen, they primarily interact with collagen through non-covalent or reversible covalent bonds. Furthermore, their application is often accompanied by color changes or the development of off-odors, which may impact consumer acceptance.

*N*-(3-Dimethylaminopropyl)-N′-ethylcarbodiimide hydrochloride (EDC) is widely recognized as a highly biocompatible cross-linking agent. When utilized as a cross-linking agent, the carboxyl group (-COOH) on the side chain of collagen initially reacts with EDC to form an O-acyl isourea intermediate, which is inherently unstable in aqueous solutions. To stabilize this intermediate, *N*-hydroxysuccinimide (NHS) is introduced, thereby enhancing the cross-linking efficacy [13,14]. This modification allows the -COOH group to react efficiently with the adjacent amino group (-NH_2_), resulting in the formation of a stable amide bond [15]. Although EDC/NHS does not directly interact with hydrogen bonds, it may enhance and stabilize the collagen structure by establishing strong covalent amide bonds both within and between molecules [16]. Consequently, a robust covalent network is formed among collagen molecules, mitigating the looseness that originally depended on hydrogen bonds. This network reduces the likelihood of hydrogen bonds breakage due to physical or chemical influences, thereby enhancing the structural rigidity of collagen [17]. Given that RSC body walls are predominantly composed of collagen and that hydrogen bonds are crucial for protein stability, structural reinforcement may be achieved through EDC/NHS cross-linking treatment [18,19,20,21,22]. Currently, EDC/NHS cross-linking has been employed to stabilize collagen in biomaterials, such as wound dressings. However, there are limited reports on the application of EDC/NHS cross-linking for the storage of RSC, and no comprehensive research has been conducted to explore the mechanism by which it inhibits the non-enzymatic degradation of RSC. It remains unclear whether the mechanism of action of EDC/NHS on collagen is primarily reparative or reinforcing, which significantly limits the effective application of EDC/NHS in the ambient-temperature preservation of marine products, including RSC.

This study evaluated the impact of varying EDC/NHS cross-linking sequences on the protective effects of cleavage of hydrogen bonds and the storage stability of RSC. The evaluation was conducted by examining textural properties, moisture content, protein degradation, protein oxidation, thermal stability, molecular docking, and structural as well as microstructural changes. Furthermore, molecular docking was employed to elucidate the potential molecular mechanisms by which EDC/NHS stabilizes the collagen structure in RSC. This study aims to elucidate the protective effect of EDC/NHS cross-linking against urea-induced collagen destabilization in RSC during room-temperature storage, thereby providing technical support for the preservation of RSC at room temperature and promoting the development of the sea cucumber industry.

## 2. Materials and Methods

### 2.1. Materials and Reagents

The RSC (60 ± 5 g) was procured from Yantai Haizhongbao Seafood Trading Center (Yantai, China) and transported to the laboratory within 1 h while maintaining a frozen state. Morpholine ethanesulfonic acid monohydrate (MES) (CAS: 145224-94-8, purity 99%), EDC (CAS: 25952-53-8, purity 98%), and NHS (CAS: 6066-82-6, purity 98%) were obtained from Aladdin Biochemical Technology Co., Ltd. (Shanghai, China). L-hydroxyproline and 2,4-dinitrophenylhydrazine (DNPH) were sourced from Macklin Biochemical Co., Ltd. (Shanghai, China). Folin-phenol reagent and bovine serum albumin (BSA) were provided by Solarbio Technology Co., Ltd. (Beijing, China). All other reagents used in this experiment were of analytical grade and supplied by Sinopharm Chemical Reagent Co., Ltd. (Shanghai, China).

### 2.2. Sample Handling

#### 2.2.1. Hydrogen Bonds Disruption Treatment

Previous studies have demonstrated that hydrogen bonds are crucial for maintaining the stability of RSC collagen. The application of 8 M urea has been shown to effectively disrupt these hydrogen bonds in RSC collagen [10]. Building on this foundation, the present study employed 8 M urea to induce hydrogen bonds disruption in RSC collagen, thereby creating an accelerated degradation model. This model enables the rapid and controllable induction of hydrogen bonds breakage, amplifying the proteolytic effects on RSC collagen. Consequently, it facilitates the evaluation of the protective effects of EDC/NHS cross-linking, with the model exhibiting qualitative similarities to the disruption of hydrogen bonds that occurs during actual storage. RSC samples were thawed, and excess moisture was removed using filter paper. The samples were then immersed in an 8 M urea solution at 4 °C for 18 h [23]. After processing, all samples were promptly rinsed multiple times with deionized water to eliminate any residual urea solution. Subsequently, they were vacuum-sealed in polyethylene terephthalate (PET) bags and stored at 25 °C for further testing. Based on the results of the preliminary experiment, this batch of RSC was stored for a duration of only 7 days, with days 0, 3, and 7 selected as representative time points for testing. This group is designated as the hydrogen bonds disruption control group (N-H group).

#### 2.2.2. EDC/NHS Cross-Linking Treatment Prior to Hydrogen Bonds Disruption

RSC samples were thawed, and excess moisture was blotted with filter paper. The samples were then immersed in a solution containing 2 g/L EDC and 0.5 g/L NHS for 4 h, following a 30 min pretreatment in a 0.1 M MES solution. Following this treatment, the samples were thoroughly washed with a 0.1 M Na_2_HPO_4_ solution for 30 min to eliminate residual MES, EDC, and NHS molecules, along with their intermediates from the system. Subsequently, the samples were repeatedly rinsed with deionized water to ensure the complete removal of any remaining solution. The cross-linking process described above occurs at the natural, unadjusted pH. Following the EDC/NHS cross-linking treatment, the hydrogen bonds in these RSC samples were disrupted as described in Section 2.2.1. Based on the results of the preliminary experiment, this batch of RSC was stored for a duration of 60 days, with days 0, 3, 7, 30, and 60 selected as representative time points for testing. All samples were then vacuum-sealed in PET bags and stored at 25 °C for subsequent testing, referred to as the Eb group.

#### 2.2.3. EDC/NHS Cross-Linking Treatment Following Hydrogen Bonds Disruption

RSC samples underwent EDC/NHS cross-linking treatment after the disruption of hydrogen bonds. This group is referred to as the Ea group. Based on the results of the preliminary experiment, this batch of RSC was stored for a duration of 60 days, with days 0, 3, 7, 30, and 60 selected as representative time points for testing. All samples from the Ea group were subsequently stored at 25 °C for further testing.

### 2.3. Determination of EDC/NHS Residues

The quantitative analysis of EDC/NHS content in cross-linked RSC was conducted following the methodology outlined in the Chinese Pharmacopoeia. RSC samples and exudate were combined with pre-chilled deionized water at a ratio of 1:3 and homogenized under ice-cold conditions. The resulting mixture was centrifuged (10,000 rpm, 15 min, 4 °C), and the supernatant was subsequently collected.

In accordance with established protocols, precisely 0.2 mL of the test sample was measured and mixed with 1.8 mL of dimethyl barbituric acid test solution, with 0.2 mL of water serving as a blank control. The mixture was allowed to stand in the dark at room temperature for 30 min, after which 2.0 mL of a 1:1 acetic acid-pyridine solution was added and mixed thoroughly. The absorbance was then measured at 599 nm using an enzyme marker (Multiskan Sky, Thermo Scientific, Waltham, MA, USA).

### 2.4. Determination of Texture Properties Analysis (TPA)

The TPA of RSC was performed to quantify key parameters, including hardness, springiness, and chewiness. This analysis utilized a physical properties tester (TMS-Pilot, Food Technology, Hampton, VA, USA), with samples prepared as 20 × 30 × 30 mm cubes. Under the specified testing conditions, a 5 mm cylindrical probe compressed the samples at a rate of 60 mm/min, achieving 60% deformation over two consecutive cycles, initiated by a trigger force of 0.03 N [10,24]. All measurements were replicated a minimum of six times.

### 2.5. Determination of Moisture Content

Moisture content was determined via gravimetric analysis, as described by Zhu et al. [25]. This procedure involved oven-drying 2 g samples at 105 ± 2 °C until a constant weight was reached, followed by calculating the percentage of weight loss.

### 2.6. Determination of Soluble Components

RSC samples and exudate were combined with pre-chilled deionized water in a 1:9 ratio and homogenized under ice-cold conditions. The mixture was centrifuged (10,000 rpm, 15 min, 4 °C), and the supernatant was collected.

Following established protocols, soluble protein (SP), free hydroxyproline (FHYP), and free ammonia nitrogen (FAN) were quantified using colorimetric methods. The SP content was determined according to the method described by Lowry et al. [26], while the analyses for FHYP and FAN adhered to the protocols outlined by Sun et al. [27] and Li et al. [28], respectively. Standard curves for FHYP and FAN quantification were constructed using hydroxyproline and glycine solutions, with absorbance values recorded at 560 nm for FHYP and 570 nm for FAN. All measurements were performed using an enzyme marker (Multiskan Sky, Thermo Scientific, Waltham, MA, USA) and conducted in triplicate.

### 2.7. Determination of Carbonyl Content

The carbonyl content was determined using the DNPH method, following the procedure outlined by Wang et al. [29] with minor modifications. RSC samples were combined with pre-chilled deionized water in a 1:3 ratio and homogenized under ice-cold conditions. The mixture was centrifuged (10,000 rpm, 15 min, 4 °C), and the supernatant was collected. A 200 μL aliquot of the sample solution was mixed with 200 μL of 10 mM DNPH and reacted at room temperature in the dark for 1 h. Subsequently, 1 mL of 20% trichloroacetic acid was added, followed by centrifugation (10,000 rpm, 15 min, 4 °C) to remove the supernatant. The resulting protein pellet was washed with 1 mL of an ethanol: ethyl acetate solution (1:1 *v*/*v*) and then centrifuged again (10,000 rpm, 15 min, 4 °C). This washing step was repeated until the yellow color of the protein precipitate disappeared. The collected pellet was then resuspended in 1 mL of 6 M guanidine hydrochloride solution and incubated at 37 °C for 15 min. Finally, the absorbance of the supernatant was measured at 370 nm, and the protein carbonyl content was calculated using a molar extinction coefficient of 22,000 M^−1^cm^−1^, expressed in micromoles per gram of protein.

### 2.8. Thermal Stability

The method for determining the thermal stability of RSC samples was adapted from the research protocol established by Bayrak et al. [30]. Specifically, 10 mg of RSC body wall tissue was placed in a sealed crucible and analyzed using a differential scanning calorimeter (µDSC III, Setaram Instrumentation, Caluire, France), which was heated from 20 to 200 °C at a rate of 10 °C/min. An empty crucible served as the control group. The enthalpy change (ΔH) of the RSC was calculated using the following formula:

(1) ΔH (J/g) = *Area*/*m*

where *Area* represents the peak area (J) and *m* denotes the mass of the sample (g).

### 2.9. Fluorescence Spectroscopy

The RSC samples were minced and thoroughly homogenized with deionized water at a 1:4 (*w*/*v*) ratio at 4 °C, using a speed of 10,000 rpm for 1 min. The resulting homogenates were shaken at 4 °C for 1 h to facilitate protein extraction. Following this shaking, the homogenates were centrifuged (10,000 rpm, 15 min, 4 °C), and the supernatants were collected, freeze-dried, and stored for subsequent analysis.

All lyophilized samples were uniformly diluted with deionized water to achieve a protein concentration of 1 mg/mL. Fluorescence intensity was measured by scanning the fluorescence spectrum using a fluorometer (Hitachi F-2700, HITACHI, Ibaraki, Japan) under the following conditions: an excitation wavelength of 280 nm and a scanning range of 310–450 nm [31].

### 2.10. Fourier Transform Infrared Spectroscopy (FTIR)

RSC samples were accurately weighed and prepared as 10% homogenates, followed by centrifugation (10,000 rpm, 15 min, 4 °C). The supernatant was collected and subjected to vacuum freeze-drying. The resulting lyophilized sample was thoroughly ground and mixed with potassium bromide (KBr) powder in a 1:100 ratio, and subsequently pressed into a pellet. This pellet was then placed in an infrared spectrometer (Nicolet iS 10, Thermo Fisher Scientific, Waltham, MA, USA) for analysis. The spectral scan range for the samples was set between 4000 and 400 cm^−1^, with a resolution of 4 cm^−1^ [32].

### 2.11. Circular Dichroism (CD) Spectroscopy

Proteins were extracted from RSC samples in each group following the method described in Section 2.8 and subsequently prepared as lyophilized pellets. Prior to measurement, the lyophilized samples from each RSC group were diluted in deionized water to achieve a concentration of 0.2 mg/mL. CD spectra were recorded using a circular dichroism spectrometer (J-1500, JASCO, Tokyo, Japan). The operational parameters were set as follows: a resolution of 1 nm, a bandwidth of 1 nm, a sensitivity of 50 nm, a response time of 2 s, and a scanning speed of 100 nm/min. Measurements were performed in the far-ultraviolet region (200–260 nm). The percentage content of each secondary structure was calculated using CNDD 2.1 software [33].

### 2.12. Scanning Electron Microscope (SEM)

The SEM workflow employed in this study was adapted from the methodology outlined by Cheng et al. [34], with minor modifications. RSC sections measuring 5 × 5 × 5 mm were fixed in a 2.5% glutaraldehyde solution at 4 °C for 24 h. Following rinsing with deionized water, the samples underwent a stepwise dehydration process using ethanol, followed by critical point drying and subsequent mounting onto metal holders. After gold coating, the specimens were observed using a SEM (JSM-7800F, JEOL, Akishima, Japan) at magnifications of 2000× and 5000×.

### 2.13. Van Gieson’s (VG) Staining

The VG staining procedure employed in this experiment adhered to the protocol established by Cheng et al. [34], with minor modifications. Samples were sectioned into blocks measuring 10 × 5 × 5 mm, fixed in 4% (*v*/*v*) formaldehyde, and subsequently dehydrated using a gradient series of ethanol. Following paraffin embedding, sections were prepared at a thickness of 7 μm per slide. After the VG staining process, the stained sections were examined under an optical microscope (CX43, OLYMPUS, Hachioji, Japan) at magnifications of 200× and 400×.

### 2.14. Molecular Docking

The three-dimensional structure of collagen from the body wall of the sea cucumber (Protein existence: Predicted, ID: A0A2G8JWP1·A0A2G8JWP1_STIJA, EC number: EC:1.14.11.4, Identifier: UP000230750, Taxonomic identifier: 307972, Organism: *Stichopus japonicus*) was retrieved from the UniProt database (https://www.uniprot.org/uniprotkb accessed on 12 March 2026). This structure was then imported into PyMOL 1.3.1 for optimization, which included the removal of water molecules, the addition of hydrogen atoms, charge assignment, and the repair of any missing amino acid residues and side chains. Subsequently, the optimized protein structure was exported in PDB format. The chemical structures of EDC and NHS were sourced from PubChem (https://pubchem.ncbi.nlm.nih.gov accessed on 12 March 2026). Hydrogen atoms and partial charges were added to each small molecule to classify them as ligands, and the rotary bonds and torsion angles of each ligand were examined. AutoDock Tools 1.5.7 was utilized to convert the PDB files of proteins and ligands into PDBQT format for molecular docking. AutoDock Vina 1.5.7 performed 50 independent docking runs to ensure statistical reliability. The final docking result selected the conformation with the highest frequency and the optimal (lowest) binding energy. Visualization of the docking results was conducted using PyMOL 1.3.1, including three-dimensional structures and surface models, to analyze the binding patterns and interactions between collagen in the sea cucumber body wall and EDC/NHS.

### 2.15. Statistical Analysis

To ensure accuracy, each treatment group at each time point utilized three biological replicates. Unless otherwise specified, all measurements were performed with a minimum of three technical replicates. For comparisons involving all three groups, including between-group comparisons and comparisons across different time points, a paired ANOVA followed by Tukey’s test was employed (*p* < 0.05). In contrast, for comparisons involving only the Eb and Ea groups, an independent samples *t*-test followed by Tukey’s test was utilized (*p* < 0.05). Pearson’s correlation analysis was employed to examine the intrinsic relationships among the various RSC parameters across the groups. Results are presented as the mean ± standard deviation (SD) derived from triplicate experiments. Statistical analyses and graphical designs were performed using SPSS 23.0 software (SPSS Inc., Chicago, IL, USA) and GraphPad Prism 10.0 software (Origin Lab Corporation, Waltham, MA, USA).

## 3. Results and Discussion

### 3.1. Residual Detection and Safety Assessment of EDC/NHS in Cross-Linked RSC

To preliminarily assess the safety of EDC/NHS cross-linked RSC, a quantitative analysis of residual EDC/NHS was performed on the treated samples. The results indicated that no detectable residues of EDC/NHS were present in any of the samples, thereby meeting the requirements stipulated the Chinese Pharmacopoeia. This finding suggests that the washing procedure utilizing 0.1 M Na_2_HPO_4_, followed by repeated rinsing with deionized water, effectively removed residual cross-linking agents from the RSC tissue, ensuring the safety of the EDC/NHS cross-linking treatment.

### 3.2. Effect of EDC/NHS Cross-Linking Treatment on the TPA of RSC with Disrupted Hydrogen Bonds

**Figure 1:**
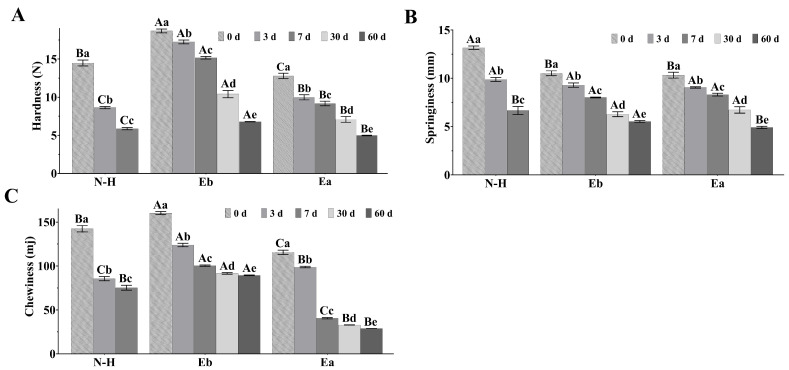
The TPA of RSC during storage at room temperature. (**A**) Hardness; (**B**) Springiness; (**C**) Chewiness. Different uppercase letters represent significant differences (*p* < 0.05) between groups on the same day, whereas different lowercase letters denote significant differences (*p* < 0.05) within groups across different days.

Changes in TPA serve as critical indicators of product quality deterioration, predicting shelf life and evaluating processing techniques and preservation effectiveness. TPA extends beyond mere texture; it directly reflects the physical and chemical changes occurring within the sea cucumber. As illustrated in Figure 1, on day 0, the hardness and chewiness in the N-H group were found to be intermediate between those of the Eb and Ea groups. However, the RSC of the N-H group displayed a translucent appearance due to the degradation of urea. Moreover, as storage time progressed, the TPA value in the N-H group deteriorated sharply, dropping to a level significantly lower than that of the EDC/NHS cross-linking groups. Notably, softening of the RSC in the N-H group was already evident by day 1 of storage, accompanied by considerable liquid exudation. By day 3 of storage, the autolysis of the RSC in the N-H group had intensified further, resulting in challenges in maintaining an intact appearance. By day 7, severe autolysis had converted the N-H group into a viscous liquid, rendering it impossible to obtain a sample for TPA value testing. Analysis indicated that urea-induced cleavage of hydrogen bonds caused acute disintegration of the RSC collagen network, thereby weakening its fundamental mechanical strength and triggering tissue autolysis. These findings confirm that hydrogen bonds are a key determinant of RSC storage stability, consistent with the earlier conclusions of Tian et al. [35]. In contrast, RSC subjected to EDC/NHS cross-linking exhibited a slower trend of quality deterioration. Due to the inhibition of autolysis, neither softening nor autolysis was observed in either group of RSC until day 30 of storage. Furthermore, samples remained available for TPA value testing up to day 60. Collectively, these findings indicate that EDC/NHS cross-linking effectively mitigates softening and autolysis of RSC during storage. In contrast to the RSC treated with Apium graveolens, as reported by Zhu et al. [28], which achieved a storage life of 30 days, the RSC treated with EDC/NHS cross-linking exhibited a significantly extended storage life of up to 60 days. This improvement occurred despite the disruption of hydrogen bonds by urea, allowing for the continued measurement of TPA data. Notably, the TPA values in the Eb group significantly surpassed those in the Ea group. This discrepancy can be attributed to the EDC/NHS cross-linking, which constructs robust covalent networks among collagen molecules. This process restricts the mobility of the molecular chains and reinforces the internal structure of collagen. Consequently, the intrinsic hydrogen bonds are stabilized, the triple-helix conformation of collagen is preserved, and its overall resistance to degradation is enhanced. Therefore, following EDC-NHS cross-linking, subsequent treatment with urea to disrupt hydrogen bonds produces a favorable protective effect. Conversely, in the Ea group, urea initially disrupts the hydrogen bonds, severely compromising the collagen structure. As a result, EDC/NHS loses its binding sites, hindering the formation of the amide bonds.

### 3.3. Effect of EDC/NHS Cross-Linking Treatment on the Moisture Content of RSC with Disrupted Hydrogen Bonds

**Figure 2:**
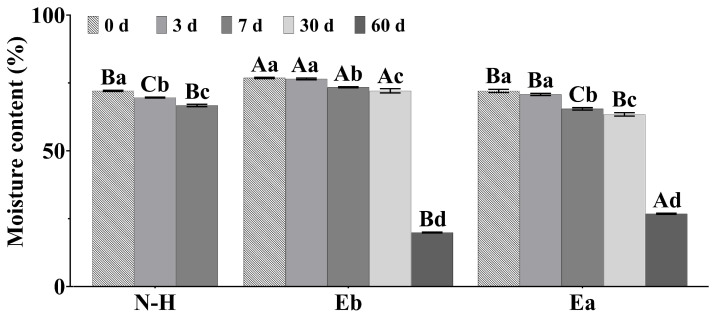
The moisture content of RSC during storage at room temperature. Different uppercase letters represent significant differences (*p* < 0.05) between groups on the same day, whereas different lowercase letters denote significant differences (*p* < 0.05) within groups across different days.

As illustrated in Figure 2, the RSC exhibited significant variations in moisture content throughout the storage period. Moisture content serves as a crucial monitoring indicator, comprehensively reflecting trends in product stability, safety, and quality. It directly influences product texture and mouthfeel and is closely associated with various chemical reactions, including the degradation of components such as proteins [36]. On day 0, the N-H group displayed the lowest moisture content, recorded at 72.09%. This finding can be attributed to urea disrupting hydrogen bonds within the collagen matrix of the RSC body wall, leading to structural instability and relaxation of the collagen framework, thereby weakening its water-holding capacity. Both the N-H and Ea groups demonstrated significant moisture loss during storage. In contrast, the Eb group exhibited a more gradual decrease in moisture content, stabilizing at 72.11% by day 30. In summary, the disruption of the hydrogen bonds network directly diminishes collagen’s water retention capacity. Mechanistically, EDC/NHS cross-linking treatment enhances structural rigidity, promoting a more compact conformation of RSC collagen. This modification provides critical structural support for water retention and confers a significant advantage [37].

### 3.4. Effect of EDC/NHS Cross-Linking Treatment on the Degradation of Proteins in RSC with Disrupted Hydrogen Bonds

**Figure 3:**
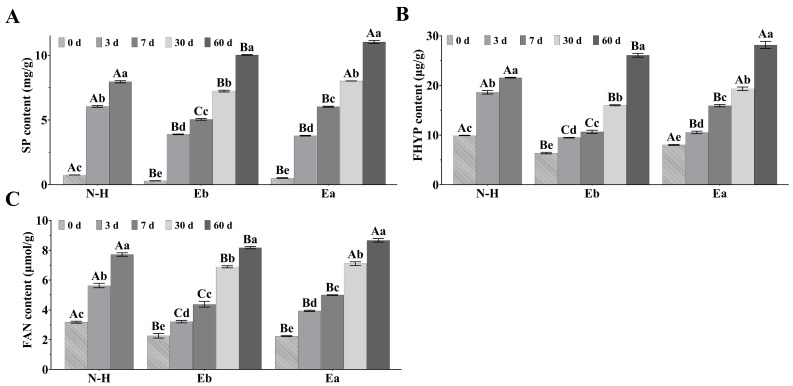
The protein degradation in RSC during storage at room temperature. (**A**) SP content; (**B**) FHYP content; (**C**) FAN content. Different uppercase letters represent significant differences (*p* < 0.05) between groups on the same day, whereas different lowercase letters denote significant differences (*p* < 0.05) within groups across different days.

Collagen constitutes approximately 70% of the proteins in the RSC body wall, and its stable triple-helix structure renders it nearly insoluble in water. However, when this triple-helix structure is disrupted, soluble components, including SP, FHYP, and FAN are released. Elevated SP concentrations directly indicate the degradation of the protein network, while increased FHYP levels serve as direct evidence of disruption in the collagen network of the RSC body wall. This disruption leads to softening and loss of elasticity in the texture of sea cucumbers, marking a critical indicator of compromised structural integrity of the proteins. Concurrently, the accumulation of FAN signifies protein breakdown and the formation of nitrogen-containing compounds. Thus, these three parameters collectively provide a multi-faceted and comprehensive analysis of the fundamental causes underlying RSC quality deterioration [38]. As illustrated in Figure 3, the impact of EDC/NHS cross-linking and hydrogen bonds disruption on protein degradation in RSC was examined. Figure 3A displayed the variation in SP content within the exudate of RSC samples from the three groups over the storage period. On day 0, SP levels in all groups were below 1 mg/g. This initial low concentration may be attributed to partial protein degradation caused by urea disrupting hydrogen bonds, leading to SP dispersion in the urea solution or loss during washing with deionized water. By day 3, SP content increased rapidly and significantly across all groups, reaching 6.04 mg/g, 3.79 mg/g, and 3.89 mg/g, respectively. Notably, the N-H group reached 7.95 mg/g as early as day 7, a value significantly higher than that observed in the EDC/NHS cross-linking groups at the same time point. SP levels in the Eb and Ea groups remained at 10.02 mg/g and 11.02 mg/g, respectively, until day 60. These findings regarding SP content revealed the crucial role of hydrogen bonds in maintaining the stability of RSC proteins. Once the hydrogen bonds network was disrupted, stable collagen fibers dissociated into soluble fragments, consistent with the findings of Tian et al. [39]. The EDC/NHS cross-linking treatment mitigated the detrimental effects of urea on intermolecular hydrogen bonds within proteins, thereby enhancing the overall structural integrity.

As a unique amino acid component of collagen, the concentration of FHYP in RSC body walls serves as an indirect indicator of collagen levels. This characteristic enables variations in FHYP concentration to effectively signify the degree of collagen fiber degradation [40]. Figure 3B illustrates the trend in FHYP content across three groups of RSC samples stored at room temperature. The FHYP levels in the N-H group were significantly higher than those in both EDC/NHS cross-linking groups. Figure 3C depicted the effects of hydrogen bonds disruption and EDC/NHS cross-linking treatment on FAN content in RSC. The N-H group exhibited significantly higher FAN content compared to the Eb and Ea groups. Research indicates that urea substantially disrupts the hydrogen bonds network within proteins, thereby accelerating their degradation process and promoting the accumulation of FHYP and FAN. This phenomenon is directly supported by the findings of Zhang et al. [41]. This discovery establishes a clear link between hydrogen bonds disruption, protein degradation, and metabolite accumulation, providing a coherent explanation for the potential mechanism by which urea contributes to RSC quality deterioration. In contrast, EDC/NHS cross-linking significantly enhances the structural rigidity of collagen, thereby indirectly delaying its degradation process. Notably, even under harsh conditions that disrupt hydrogen bonds, induced by urea, the EDC/NHS-treated RSC maintained FHYP levels below 30 μg/g after 60 days of storage. This outcome is favorable when compared to the degradation extent reported by Peng et al. [9] for high-pressure steam-sterilized sea cucumbers stored for 33 days, where FHYP increased from 8.33 to 24.12 μg/g. This comparison suggests that EDC/NHS cross-linking effectively suppresses collagen degradation under milder reaction conditions.

### 3.5. Effect of EDC/NHS Cross-Linking Treatment on the Degree of Protein Oxidation and Tertiary Structure of RSC with Disrupted Hydrogen Bonds

Protein oxidation is primarily associated with the formation of protein radicals, which can lead to protein fragmentation, functional impairment, and quality deterioration. Carbonyl compounds are generated through the oxidative deamination of basic amino acids, such as lysine (Lys) and arginine (Arg). Consequently, the quantitative detection of protein carbonyls serves as a reliable indicator for assessing the degree of protein oxidation [42].

**Figure 4:**
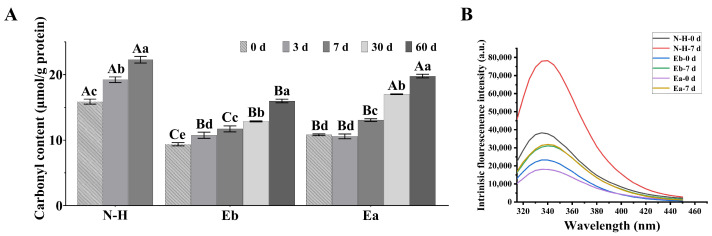
The degree of protein oxidation of RSC during storage at room temperature. (**A**) Carbonyl content; (**B**) Fluorescence spectroscopy. Different uppercase letters represent significant differences (*p* < 0.05) between groups on the same day, whereas different lowercase letters denote significant differences (*p* < 0.05) within groups across different days.

As illustrated in Figure 4A, the carbonyl content in the N-H group exhibited a rapid increase, reaching 22.27 μmol/g protein on the seventh day of storage at room temperature. In contrast, the Eb and Ea groups displayed carbonyl contents of only 12.50 μmol/g protein and 16.53 μmol/g protein, respectively, by day 30. This finding suggests that urea molecules disrupt the hydrogen bonds interactions that stabilize the secondary and tertiary structures of proteins. Such disruption results in the unwinding and unfolding of the triple-helix structure, resulting in a loss of mechanical strength and function. Consequently, sensitive amino acid side chains and peptide backbone structures in RSC collagen become exposed. During storage, exposure to oxygen and pro-oxidative factors initiates free radical oxidation reactions, which oxidize sensitive amino acid side chains (e.g., Lys, Arg), leading to the generation of aldehyde-containing derivatives and a significant increase in detectable carbonyl content. However, EDC/NHS cross-linking establishes a stable three-dimensional network, effectively suppressing the increase in carbonyl content. Notably, the Eb group exhibited a significantly lower carbonyl content compared to the Ea group, maintaining a low level of 15.52 μmol/g protein even after 60 days of storage. In contrast, the carbonyl content of the Ea group on day 60 was 19.22 μmol/g protein. This phenomenon may be attributed to the protective effect of EDC/NHS cross-linking, which is conducted prior to the disruption of hydrogen bonds by urea. This process enhances the structural rigidity of collagen and reduces the exposure of regions within the collagen that are susceptible to oxidation [43].

Tryptophan (Trp), a typical oxidation-sensitive amino acid and a prominent endogenous fluorescent chromophore in proteins, serves as an effective probe for assessing the degree of protein oxidation. In its native conformation, the Trp residue is embedded within the hydrophobic core of the protein, with its emission peak typically ranging from 330 to 350 nm. Upon denaturation and the disruption of the protein’s tertiary structure, Trp residues may become exposed to a more hydrophilic environment. This exposure typically results in a red shift in the maximum emission peak, which shifts to 350–360 nm. The accompanying changes in fluorescence intensity may vary depending on the local environment and the oxidation state. This shift indicates a heightened susceptibility of the protein to oxidation. Consequently, by monitoring the emission peak of Trp via fluorescence spectroscopy, one can effectively analyze the extent of protein oxidation [44]. As illustrated in Figure 4B, after 7 days of storage, the fluorescence intensity of the N-H group significantly increased, accompanied by a noticeable red shift. This observation suggests that the disruption of the hydrogen-bond network weakened the collagen triple-helix structure, thereby exposing previously buried tryptophan residues to the polar solvent environment. Such exposure substantially increases the likelihood of collagen oxidation, which aligns closely with the sharp rise in carbonyl content of the N-H group depicted in Figure 4A.

In the Eb group, fluorescence intensity exhibited a negligible increase after 7 days of storage, with no significant red shift in the emission peak; the spectrum remained nearly identical to that recorded on day 0. This observation indicates that EDC/NHS plays a crucial role in maintaining the integrity of hydrogen bonds, thereby constructing a supportive network for collagen through the formation of stable covalent amide bonds. This mechanism effectively preserves the original hydrogen bonds network and tertiary structure, preventing urea molecules from attacking the collagen hydrogen bonds network and allowing Trp and other oxidation-sensitive amino acid residues to remain embedded within the hydrophobic core. The close correlation between the fluorescence data and carbonyl content further confirms that the oxidation-sensitive amino acids are effectively protected. In contrast, the decrease in fluorescence intensity in the Ea group was moderate, falling between the N-H group and the Eb group. This suggests that once urea disrupts the hydrogen bonds, leading to a loosening of the collagen structure, even subsequent covalent cross-linking by EDC/NHS cannot fully restore the damaged tertiary structure, leaving some Trp and other oxidation-sensitive amino acid residues exposed. The fluorescence profile of the Ea group starkly contrasts with that of the Eb group, further confirming that the optimal timing for EDC/NHS intervention is when hydrogen bonds remain intact. Its function is primarily protective rather than constructive concerning the hydrogen bonds network.

### 3.6. Effect of EDC/NHS Cross-Linking Treatment on the Thermal Stability of Collagen in RSC with Disrupted Hydrogen Bonds

**Figure 5:**
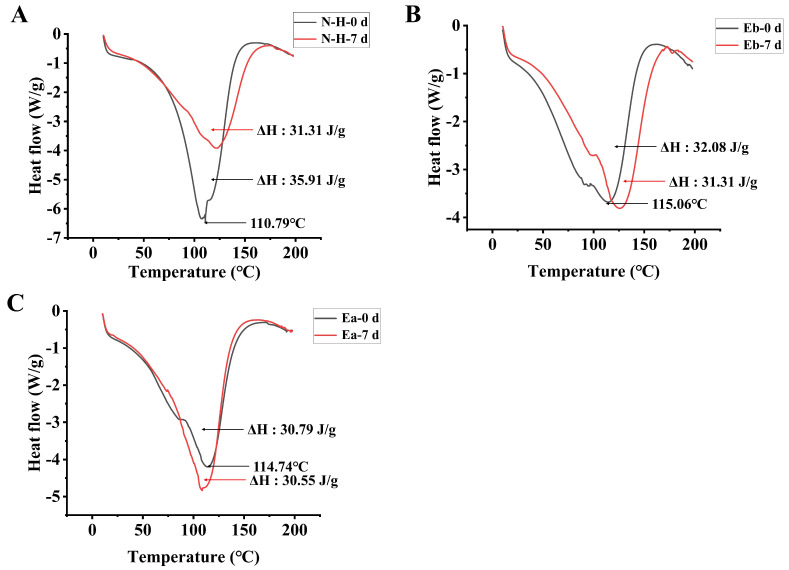
Thermal stability of collagen in RSC across different groups at both 0 and 7 days. (**A**) N-H group; (**B**) Eb group; (**C**) Ea group.

Figure 5 displayed the DSC curve of the RSC during storage. The peak area of the scan curve corresponds to the enthalpy change value (ΔH), which quantitatively reflects the stability of the chemical bonds within the RSC sample. During thermal denaturation, the proteins in the sample experience unfolding and disassembly of their naturally ordered three-dimensional structures, resulting in a disordered, randomly coiled state. The disruption of noncovalent interactions, primarily hydrogen bonds, that maintain the native structure of collagen necessitates heat absorption, manifesting as an endothermic peak on the DSC curve [45].

As illustrated in Figure 5, following hydrogen-bond-breaking treatment, the peak area of the RSC on day 7 was significantly reduced compared to day 0, and the peak shape exhibited substantial changes, with severe broadening observed on day 7. Further calculations indicate that the ΔH value of the N-H group decreased by 4.6 J/g between day 0 and day 7. Preliminary findings suggest that the non-covalent interactions responsible for maintaining the structural integrity of collagen in RSC are gradually disrupted by urea, with the extent of this disruption increasing over time, leading to a significant reduction in the structural strength of the collagen. In contrast, the thermal denaturation temperatures of the two groups of RSC treated with EDC/NHS cross-linking were significantly increased, providing direct evidence of the enhanced structural strength of the collagen. Simultaneously, the change in ΔH values for the two EDC/NHS cross-linked groups during storage was negligible, with decreases of less than 1 over a 7-day period. This finding suggests that EDC/NHS treatment enhances the structural rigidity of the collagen matrix and delays its degradation during storage. Furthermore, the changes in peak shapes indicate that the degree of peak overlap between the Eb and Ea groups before and after storage was significantly higher than that of the N-H group. This phenomenon may be attributed to the cross-linking mechanism of EDC/NHS, whereby the covalent amide bonds formed reinforce the structural rigidity of the collagen, enabling it to withstand the damaging effects of urea and retain greater integrity during storage. It is noteworthy that on day 0, group Eb (115.06 °C, 32.08 J/g) exhibited a higher thermal denaturation temperature and ΔH value compared to group Ea (114.74 °C, 30.79 J/g). This observation suggests that the amide bond network established through the pre-crosslinking treatment effectively protects collagen from degradation by urea. This finding partially underscores the role of EDC/NHS in reinforcing and safeguarding the collagen structure.

### 3.7. Effect of EDC/NHS Cross-Linking on Protein Secondary Structures of RSC with Disrupted Hydrogen Bonds

FTIR spectroscopy was employed to investigate the effects of various treatments on the protein structure in RSC samples. Collagen, the primary component of RSC, demonstrates structural stability in its triple-helix configuration, which is intricately associated with hydrogen bonds interactions. In contrast to macroscopic indicators, FTIR spectroscopy offers deeper insights into the disruption of the internal hydrogen bonds network within collagen’s triple-helix and captures the transition trends towards disordered coiled or β-folded states. Consequently, FTIR can provide valuable insights into the secondary structure of collagen, thereby indirectly indicating alterations in this structure that are associated with the integrity of hydrogen bonds [46].

**Figure 6:**
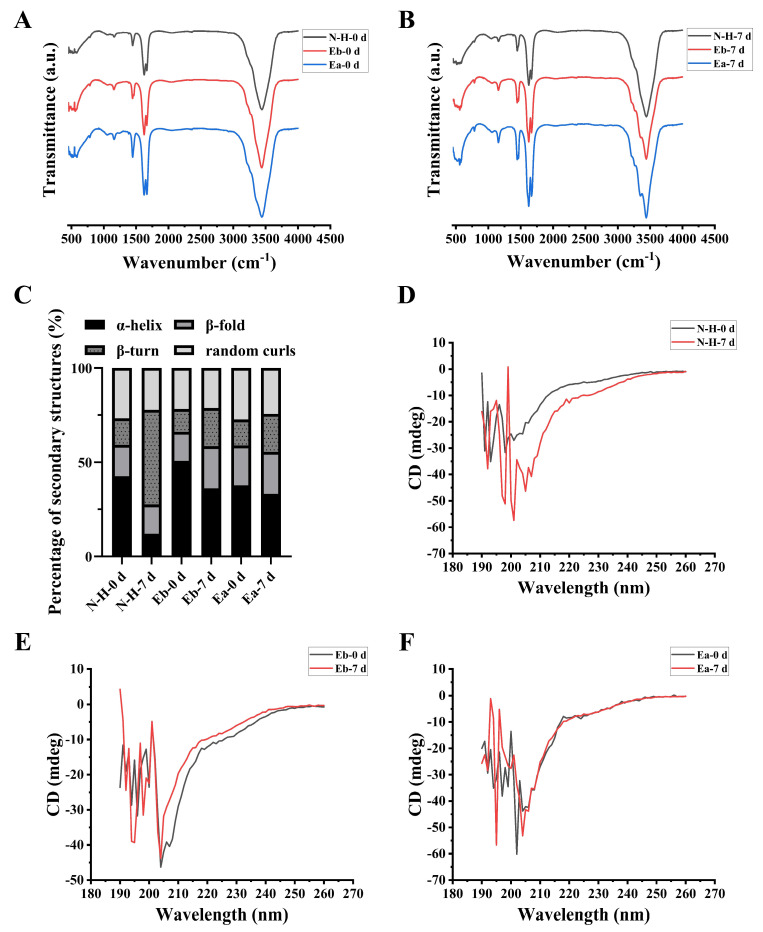
The secondary structure of collagen in RSC across different groups during the storage period. (**A**) FTIR at day 0; (**B**) FTIR at day 7; (**C**) Changes in the proportion of secondary structures across groups; (**D**) CD of the N-H group; (**E**) CD of the Eb group; (**F**) CD of the Ea group.

Figure 6A,B displayed the FTIR spectra of RSC samples subjected to different treatments and stored at 25 °C. Previous studies have indicated that a shift in the amide I band toward higher wavenumbers signifies a reduction in hydrogen bonds within collagen, leading to compromised structural integrity. The N-H group exhibits a higher wavenumber (1628–1670 cm^−1^) in the oscillating amide I band, accompanied by a weak -OH peak [10]. This phenomenon indicates that the ordered structure of collagen has been disrupted, resulting in an increase in structural disorder. Conversely, the Eb and Ea groups induce a shift in the amide I band to a lower wavenumber, suggesting an enhancement in the stability of the collagen secondary structure network [47]. This effect may be attributed to the mechanism of EDC/NHS cross-linking: EDC activates the -COOH group on the side chain of collagen to form an unstable intermediate, which is then stabilized by NHS to react efficiently with the adjacent -NH_2_, resulting in the formation of an amide bond. This reaction leads to a shift in the amide I band to a lower wavenumber. Simultaneously, the binding capacity of the collagen network to water is enhanced following EDC/NHS cross-linking, resulting in a pronounced broad peak of -OH stretching vibration in the FTIR spectrum.

Figure 6C provides a clearer illustration of the alterations in the secondary structure of the RSC across each group during storage. As depicted in Figure 6C, the triple-helix integrity structure content in the N-H group decreased markedly by 30.48% during storage, while the disordered conformation content increased significantly by 69.52%. This phenomenon indicates that the breakdown of the hydrogen-bond network facilitated a transition from the ordered triple-helix integrity structure to the disordered conformation within the secondary structure, which serves as the molecular basis for the overall deterioration of the N-H group concerning macroscopic quality indicators. In contrast, the Eb group exhibited a substantially higher proportion of α-helices than the N-H group both before and after storage. This observation suggests that the covalent amide bond framework effectively reinforced the natural conformation of collagen, allowing it to better withstand the damaging effects of urea and delay the onset of secondary structure disorder. It is noteworthy that, although there was no significant change in the proportion of secondary structure in the Ea group before and after storage, it remained significantly inferior to that of the Eb group. This discrepancy may arise from the initial disruption of hydrogen bonds, even with subsequent cross-linking measures, the damage to the secondary structure may have already become irreversible.

CD spectroscopic analysis provided evidence supporting the protective effect of EDC/NHS on the secondary structure integrity of RSC collagen against disorder. As illustrated in Figure 6D–F, after 7 days of storage, the CD spectrum of the N-H group exhibited significant alterations. The CD curve on day 0 displayed a weak negative peak intensity in the 190–200 nm range, approximately −35 mdeg. In contrast, the CD curve on day 7 revealed multiple sharp and pronounced negative peaks within the same region, with the maximum negative peak intensity reaching approximately −58 mdeg. Furthermore, the CD spectrum on day 7 appeared considerably more disordered overall compared to that on day 0. This transformation suggests that the hydrogen-bond-breaking action of urea induces the dissociation of collagen’s secondary structure, leading to a shift from a relatively ordered state to randomly coiled or misfolded β-turns [48].

In contrast, the CD spectra of the Eb group after 7 days of storage were virtually identical to those of the 0-day samples, exhibiting the lowest degree of spectral distortion. This phenomenon suggests that the covalent amide bond framework formed through the introduction of EDC/NHS, while preserving hydrogen bonds, effectively protected the natural secondary structure conformation of collagen by enhancing its structural stability. Conversely, the changes in the CD spectrum of the Ea group were intermediate between those of the N-H and Eb groups: although the overlap between the CD curves on day 7 and day 0 in the Ea group was better than that in the N-H group, a significant deviation remained when compared to the Eb group. This indicates a slight alteration in the secondary structure conformation of the Ea group, accompanied by an overall increase in disorder. This may be attributed to the initial disruption of hydrogen bonds by urea, leading to the dissociation of collagen’s secondary structure. Although subsequent EDC/NHS cross-linking can form amide bonds to stabilize parts of the structure, it cannot fully restore the damaged secondary structure. After 7 days of storage, the conformation of the secondary structure became further relaxed, resulting in a slight shift in the CD spectrum.

### 3.8. Effect of EDC/NHS Cross-Linking Treatment on the Microstructure of RSC with Disrupted Hydrogen Bonds

**Figure 7:**
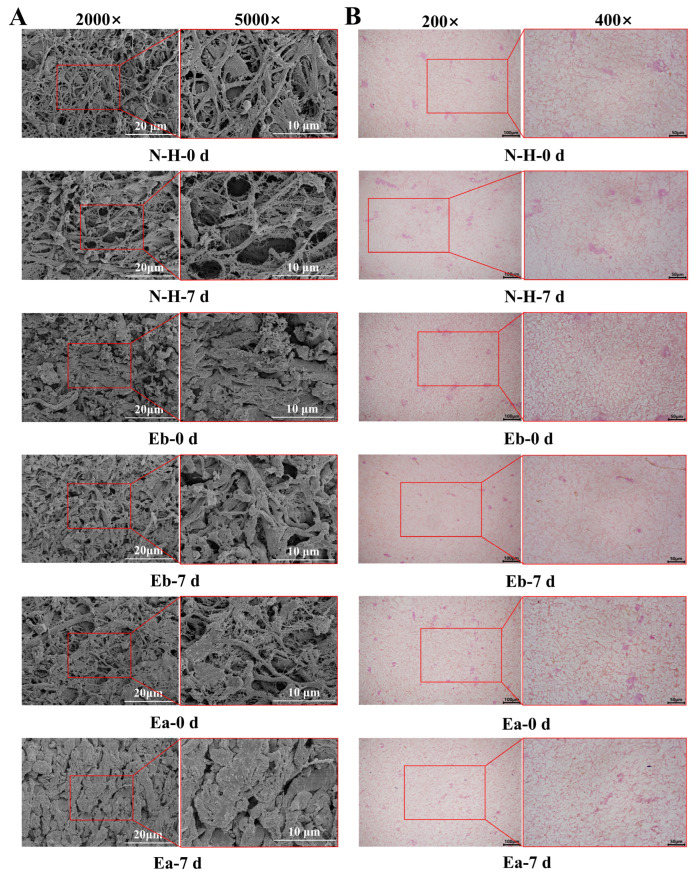
The microstructure of collagen in RSC. (**A**) SEM images at magnifications of 2000× and 5000×; (**B**) VG images at magnifications of 200× and 400×.

Figure 7A presented SEM images of RSC samples stored at 25 °C after being treated with various methods. The images clearly demonstrated that over the 7-day storage period, the gaps between collagen fibers in the N-H group’s RSC samples significantly widened, resulting in an overall loose and porous appearance. This structural degradation likely resulted from urea disrupting the hydrogen bonds that stabilize the collagen structure, ultimately compromising its structural integrity and accelerating deterioration with prolonged storage [49]. In contrast, the collagen fiber microstructure in both EDC/NHS cross-linking groups remained denser. Notably, the protective effect of EDC/NHS cross-linking on collagen fiber structure appears to surpass that of the conventional high-temperature, high-pressure treatment reported by Liu et al. [47]. In their study, although high-temperature, high-pressure processing extended the shelf-life of sea cucumbers, the extreme thermal conditions inevitably resulted in the fragmentation and loosening of collagen fibers. In contrast, EDC/NHS cross-linking effectively preserves fiber integrity under milder reaction conditions. The covalent amide network formed by EDC/NHS enhances the structural rigidity of the collagen matrix, thereby maintaining a dense arrangement of fibers. Notably, although the microstructure of the Ea group on day 7 did not exhibit a porous and loose morphology, it displayed a foamy structure. This phenomenon reflects the transition of the RSC body wall gel network from an ordered to a disordered state. As storage time extended, collagen degradation diminished the network’s structural integrity, leading to the redistribution or localized accumulation of water. This indicates that the quality of RSC in the Ea group remained inferior to that in the Eb group. This difference likely arises from the fact that the EDC/NHS cross-linking, conducted prior to the disruption of hydrogen bonds, stabilized the collagen structure and, to some extent, mitigated the subsequent damage to the hydrogen bonds caused by urea.

VG staining, a classic method specific for collagen fiber visualization, allows for a direct assessment of changes in the morphology, distribution, and integrity of the collagen fiber network within the RSC body walls during storage [50]. Figure 7B illustrated the morphological alterations in RSC tissue following VG staining. In comparison to the two EDC/NHS cross-linking groups, the N-H group exhibited significant deterioration in tissue morphology and collagen fiber structural integrity, characterized by markedly larger pores than those observed in the Ea and Eb groups. With prolonged storage, the interstitial spaces between collagen fibers in the N-H group significantly widened, while the lengths of the fibers noticeably shortened. This phenomenon is attributed to urea disrupting hydrogen bonds within the collagen fibers, leading to fiber breakage and degradation. Conversely, the Eb group displayed more tightly arranged collagen fibers with smaller interstitial spaces and more intact fiber states. These results indicated that EDC/NHS cross-linking treatment may partially inhibit the degradation of RSC body walls, thereby slowing structural damage to collagen fibers. Furthermore, the collagen fiber density in the Ea group was significantly lower than that in the Eb group, with the fibers appearing slightly thinner and shorter compared to those in the Eb group. This further confirms the protective effect of EDC/NHS cross-linking on the disruption of hydrogen bonds.

### 3.9. Elucidation of the Mechanism of Interaction Between Urea and EDC/NHS with RSC Collagen

**Figure 8:**
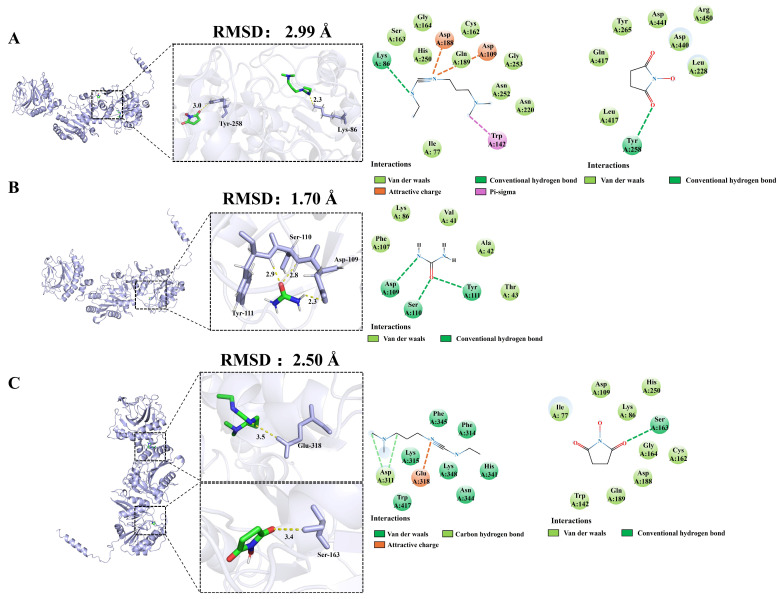
Molecular docking interactions between urea and EDC/NHS with RSC collagen. (**A**) EDC/NHS and RSC collagen; (**B**) Urea and RSC collagen; (**C**) EDC/NHS and RSC collagen following the disruption of hydrogen bonds by urea.

Molecular docking analysis predicted potential alterations in binding sites at the atomic level resulting from EDC/NHS cross-linking, both prior to and after the disruption of the collagen hydrogen bonds network by urea. This observation offers a structural explanation that complements the experimental findings. It is important to note that molecular docking is specifically designed to predict noncovalent interactions and cannot simulate the covalent reaction chemistry of EDC/NHS. Consequently, the results obtained from molecular docking provide only potential structural insights, while the actual cross-linking mechanism is corroborated by both molecular docking models and experimental data. As illustrated in Figure 8A, when the hydrogen bonds are intact, EDC molecules are precisely positioned at -COOH-rich regions of collagen, such as Asp-109 and Asp-188, through van der Waals forces and electrostatic interactions, serving as direct targets for amide bond formation. Furthermore, pi-sigma and other interactions enhance the specificity and stability of the resulting amide bond. NHS further increases the specificity and stability of the amide bond through hydrogen bonds with Tyr-258 and electrostatic interactions with Asp-441, Leu-262, and other residues. Figure 8B illustrates a molecular docking model depicting the interaction between the urea molecule and collagen. The docking results indicate that urea can form multiple hydrogen bonds with several polar residues, including Ser-110, Tyr-111, and Asp-109. Given that Zhang et al. [10] have demonstrated that urea disrupts hydrogen bonds, it can be inferred that urea may interfere with the existing hydrogen bond network by competitively binding to these residues [51].

As demonstrated in Figure 8C, the disruption of the hydrogen-bond network by urea allows EDC to interact with exposed residues such as Phe-314, Phe-345, and Asp-311 through van der Waals forces and carbon- hydrogen bonds. Notably, Glu-318 was the only remaining residue capable of forming attractive charge-specific interactions. This suggested that, following the disruption of hydrogen bonds, EDC/NHS is unable to localize to the -COOH reaction site. Furthermore, the failure to reestablish the disrupted hydrogen bonds network leads to an irreversible compromise in the order and integrity of the collagen structure. This observation indirectly elucidates why the quality parameters for the Eb group are significantly superior to those of the Ea group.

### 3.10. Correlation Analysis Among Various Parameters

**Figure 9:**
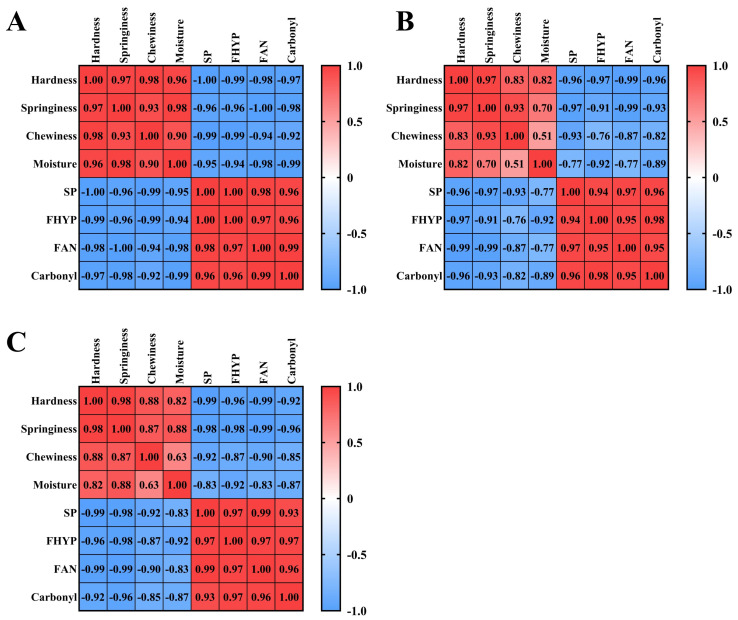
Correlation analysis of key indicators across groups. (**A**) N-H group; (**B**) Eb group; (**C**) Ea group.

To investigate the intrinsic relationships among various quality parameters and to determine whether the observed variations are mechanistically linked, this study conducted a Pearson correlation analysis. As illustrated in Figure 9A, parameters such as TPA and moisture content exhibited a significant negative correlation with the content of soluble components and carbonyl content. This finding indicates that collagen degradation is the direct cause of the softening of RSC texture, supporting the hypothesis that the disruption of hydrogen bonds leads to the depolymerization of collagen fibers. Additionally, as shown in Figure 9B,C, EDC/NHS cross-linking treatment—particularly in the Eb group—significantly decreased the correlation between soluble components and apparent RSC indices. For instance, the correlation between SP content and moisture content in the Eb group was −0.77, significantly lower than that in the N-H group (−0.95). Similarly, the correlation between FHYP content and chewability was −0.76, which was also significantly lower than that of the N-H group (−0.99). These findings indicate that pre-crosslinking disrupts the direct link between degradation and textural deterioration; even if some collagen degradation occurs during storage, the texture remains partially preserved. Notably, the trend in changes in correlation was significantly greater in the Eb group than in the Ea group, confirming the inference from earlier experiments that the Eb group was more effective than the Ea group in delaying the deterioration of RSC quality.

## 4. Conclusions

This study investigated the relationship between hydrogen bonds and the stability of RSC, along with the protective effect of EDC/NHS cross-linking treatment on the quality deterioration of RSC. The results indicated that hydrogen bonds were a key factor in maintaining the structural stability of RSC collagen. RSC treated with urea exhibited rapid deterioration across all quality parameters within 7 days of storage, characterized by a significant increase in collagen degradation and oxidation. EDC/NHS cross-linking effectively enhances the structural rigidity of collagen, thereby counteracting the quality deterioration caused by the breakdown of hydrogen bonds. Compared to the N-H group, both EDC/NHS cross-linking groups demonstrated significant improvements in all quality parameters during storage: the rate of decline in TPA parameters slowed, water-holding capacity increased, the upward trend in soluble components was suppressed, carbonyl accumulation was delayed, DSC thermal stability was well maintained, CD spectra indicated that the order of the secondary structure was preserved, fluorescence spectra confirmed a stronger integrity of the tertiary structure, and microstructural observations revealed a denser and more intact collagen fiber network. Additionally, the processing sequence has a decisive impact on the protective efficacy of EDC/NHS, with all parameters for the Eb group being significantly superior to those of the Ea group. Molecular docking simulations have further suggested the mechanism by which EDC/NHS enhances the structural stability of collagen at the atomic level. By forming covalent amide bonds on the main chain, this process reduces the overall molecular mobility of collagen and increases its structural rigidity. These findings elucidate the cross-linking mechanism of EDC/NHS and its interactions with collagen, thereby providing a significant theoretical foundation and practical guidance for room temperature preservation technologies applicable to RSC and other collagen-based aquatic products. It is important to note that this study did not include a control group of untreated RSC. Consequently, the experimental design does not permit direct inferences regarding the absolute efficacy of EDC/NHS cross-linking in comparison to untreated sea cucumbers. Therefore, the findings of this study should be regarded solely as proof of concept concerning the underlying mechanism. Future research should integrate untreated RSC as a baseline control to evaluate the preservation efficacy and practical application potential of EDC/NHS treatment. Furthermore, subsequent studies are anticipated to validate the long-term protective effects of pre-crosslinking under actual room-temperature storage conditions. These studies will also include evaluations of microbiological safety and sensory quality, thereby enhancing the feasibility of applying EDC/NHS in food processing and production.

## Data Availability

The original contributions presented in this study are included in the article. Further inquiries can be directed to the corresponding authors.

## Abbreviations

The following abbreviations are used in this manuscript:

EDC	*N*-(3-dimethylaminopropyl)-N′-ethylcarbodiimide hydrochloride
NHS	*N*-hydroxysuccinimide
MES	Morpholine ethanesulfonic acid monohydrate
RSC	Ready-to-eat sea cucumber
N-H group	Hydrogen bonds disruption control group
TPA	Texture properties analysis
SP	Soluble protein
BSA	Bovine serum albumin
DNPH	2,4-dinitrophenylhydrazine
FHYP	Free hydroxyproline
FAN	Free ammonia nitrogen
DSC	Differential scanning calorimetry
FTIR	Fourier transform infrared spectroscopy
CD	Circular dichroism
SEM	Scanning electron microscope
VG	Van Gieson
ANOVA	Analysis of variance

## References

[1] Ellis C., Elston D.M., Joustra J.P.L., Haddad V.J.R. (2021). Aquatic Antagonists: *Sea cucumbers* (*Holothuroidea*). Cutis.

[2] Yu J.Q., Ge W.H., Wang K.F., Hao W.H., Yang S.J., Xu Y., Sun X. (2024). Cross-linking Ability of Hydrolyzed Distarch Phosphate and Its Stabilizing Effect on Rehydrated *Sea cucumber*. Food Chem..

[3] Mao Y.Y., Dong Y., Xiang Y., Huang L.J., He Q. (2025). Effect of Curdlan on Improving the Property Deterioration of Fish Gelatin Gels Induced by High Temperature: An Exploration of Gel Properties, Structure and Interaction Mechanism. Food Hydrocoll..

[4] Yoon J.L., Myung J.K., Ji S.K., Seok N.K., Sang H.L. (2025). Ecological Insights into the Extinct Korean Sea Lion (*Zalophus japonicus*) in Korea Based on Stable Isotope Analysis of Bone Collagen. Rapid Commun. Mass Spectrom. RCM.

[5] Jiang J.W., Gao S., Zhao Z.L., Chen Z., Zhang F.F., Li L., Zhou Z.C. (2023). A Novel Short-type Peptidoglycan Recognition Protein with Unique Polysaccharide Recognition Specificity in *Sea cucumber*, *Apostichopus japonicus*. Fish Shellfish Immun..

[6] Li X.N., Tian Y., Xiao H.R., Tian F.L., Han L.S., Zhao C., Ding J. (2025). Compound Inhibitors Mitigate Skin Ulceration Induced by UVA and *Vibrio splendidus* in the *Sea cucumber Apostichopus japonicus*. Fishes.

[7] Amit D., Abul H., Deepika D. (2024). The Effect of Pre-Treatment and the Drying Method on the Nutritional and Bioactive Composition of *Sea cucumbers*—A Review. Appl. Sci..

[8] Chen T.J., Peng Z., Lu J.H., Li B.F., Hou H. (2016). Self-Degradation of *Sea cucumber* Body Wall Under 4C Storage Condition. J. Food Process. Preserv..

[9] Peng Z., Hou H., Bu L., Li B.F., Zhang Z.H., Xue C.H. (2015). Nonenzymatic Softening Mechanism of Collagen Gel of *Sea cucumber* (*Apostichopus japonicus*). J. Food Process. Preserv..

[10] Zhang C.P., Li L.H., Wang Q.T., Xie Y.L., Gao J.R., Li M.B., Sun L.L. (2024). Effect of Thermal Treatment and Secondary Bonds on the Storage Stability of Ready-to-eat *Sea cucumbers*. Process. Biochem..

[11] Zhu L.L., Qi X., Bai J., Sun X., Hou H. (2022). The Mechanism of Molecular Cross-linking Against Nonenzymatic Degradation in the Body Wall of Ready-to-eat *Sea cucumber*. Food Chem..

[12] Yu M., Wang Y.Z., Xie Y.Q., Dong X.F., Nakamura Y., Chen X., Qi H. (2023). Polyphenol Extracts from Ascophyllum Nodosum Protected *Sea cucumber* (*Apostichopus japonicas*) Body Wall Against Thermal Degradation During Tenderization. Food Res. Int..

[13] Sara B., Farooq A., Nazamid S. (2011). High-Value Components and Bioactives from *Sea cucumbers* for Functional Foods—A Review. Mar. Drugs.

[14] Şule A., Ali K., Duygu E. (2024). CMC/Gel/GO 3D-printed Cardiac Patches: GO and CMC Improve Flexibility and Promote H9C2 Cell Proliferation, while EDC/NHS Enhances Stability. Biofabrication.

[15] Carson C., Brett H.P., Vardan H.V., Kiana A.C., Thi H.B., Adam C.F., Jeffrey D.H. (2025). Covalent Stabilization of Collagen Mimetic Triple Helices and Assemblies by Dopa Crosslinking. Chembio.

[16] Liao Z.L., Yu H., He G.H., Ruan X.H., Li H.L., Dou J.H., Zhang X.J. (2025). Carboxymethyl Chitosan Modification Via EDC/NHS-mediated Amidation for Performance Improvement on Blood-contacting Biomedical Membranes. Int. J. Biol. Macromol..

[17] Gong H., Zi Y., Kan G., Li L., Shi C., Wang X.C., Zhong J. (2024). Preparation of Food-grade EDC/NHS-crosslinked Gelatin Nanoparticles and Their Application for Pickering Emulsion Stabilization. Food Chem..

[18] Gu W.W., Gu L., Tao N.P., Wang X.C., Xu C.H. (2025). Composite Fish Collagen Peptide-Based Biopolymer Emulsion for Keratin Structure Stabilization and Hair Fiber Repair. Polymers.

[19] Xu F.C., Li H.L., Li Y. (2024). *Sea cucumber*-Inspired Polyurethane Demonstrating Record-Breaking Mechanical Properties in Room-Temperature Self-Healing Ionogels. Adv. Mater..

[20] Angela M.R.G., Marco A.A.P., Filiberto R.T., María C.P.B. (2025). Study of the Effect of Two Different Chemical Cross-Linking Agents (EDC/NHS and Genipin) on the Physical, Chemical, and Mechanical Properties of Collagen, Polycaprolactone, and Chitosan Scaffolds. Materials.

[21] Lee B., Kim J.Y., Choi Y.M. (2025). Sous-vide Treatment Strategies for Enhancing Quality Traits in Various Meat Products: Physicochemical, Organoleptic, and Microbiological Perspectives. Appl. Food Res..

[22] Liang J.M., Wang B., Zhang J.M., Bai T., Zhong Z.G., Tang Z.H. (2025). Bioprotective Potential of Lactic Acid Bacteria in Pickled Pepper Rabbit Meat During Refrigerated Storage. Foods.

[23] Yu N.N., Xu Y.S., Jiang Q.X., Xia W.S. (2017). Molecular Forces Involved in Heat-induced Freshwater Surimi Gel: Effects of Various Bond Disrupting Agents on the Gel Properties and Protein Conformation Changes. Food Hydrocoll..

[24] Qin K.M., Sun X.Y., Liu J.M., Wang R.C., Huang X.F., Wang Y., Wang S. (2025). A Rosmarinic Acid-Fish Skin Protein-chitosan Hybrid Nano-delivery System with Excellent Sustained-release and Antioxidant Performances. Food Chem..

[25] Zhu Y.Y., Zhu J.Y., Shi X.B., Fan M.D. (2025). Effects of Freezing, Frozen Storage and Thawing on the Water Status, Quality, Nutrition and Digestibility of Meat: A Review. Food Sci. Nutr..

[26] Lowry O., Rosebrough N., Farr A., Randall N. (1951). Protein Measurement with the Folin Phenol Reagent. J. Biol. Chem..

[27] Sun X., Zhu L.L., Qi X., Zhang H.W., Wu L., Wang J.H., Hou H. (2021). Cleavage sites and non-enzymatic self-degradation mechanism of ready-to-eat *Sea cucumber* during storage. Food Chem..

[28] Li X.M., Luo L., Cai Y., Yang W.J., Lin L.S., Li Z., Zhao J. (2017). Structural Elucidation and Biological Activity of a Highly Regular Fucosylated Glycosaminoglycan from the Edible *Sea cucumber Stichopus herrmanni*. J. Agric. Food Chem..

[29] Wang W.X., Guan W.Q., Zhu M., Cui L.Y., He X.X., Song Y., Zhang H. (2025). Effects of Continuous High-voltage Alternating Electric Field Treatment on the Color, Lipid Oxidation, and Myofibrillar Protein of Pork During Cold Storage. J. Food Sci..

[30] Bayrak S. (2025). Immobilized Black Radish Peroxidase on ZnO-alginate Beads: A Multifunctional Biocatalyst for Sustainable Food Packaging. Food Sci. Nutr..

[31] Matiacevich S.B., Buera M.P. (2005). A critical evaluation of fluorescence as a potential marker for the Maillard reaction. Food Chem..

[32] Cui S., Sun M.J., Yang J.H., Zhang D., Liu K., Tao H., Zhao C.Q. (2025). Nano-chitosan-nisin Composite Membrane: Physicochemical Properties and Its Impact on Sturgeon Storage Quality. LWT.

[33] Yan J., Shang W., Zhao J., Han J., Jin W. (2019). Gelation and Microstructural Properties of Protein Hydrolysates from Trypsin-treated Male Gonad of Scallop (*Patinopecten yessoensis*) Modified by κ-Carrageenan/K^+^. Food Hydrocoll..

[34] Cheng Y.C., Jin D.L., Yu W.T., Tan B.Y., Fu J.J., Chen Y.W. (2024). Impact of Thermal Ultrasound on Enzyme Inactivation and Flavor Improvement of *Sea cucumber* Hydrolysates. Food Chem..

[35] Tian Q.J., Lin L., Qi X., Zhu L.L., Hao L., Wu L., Hou H. (2022). Contribution of Secondary Bonds to the Storage Stability of Ready-to-eat *Sea cucumber*. Food Chem..

[36] Dankar I., Amira H., Montserrat P., Francesc S. (2024). Hydrogen bonds Integration in Potato Microstructure: Effects of Water Removal, Thermal Treatment, and Cooking Techniques. Polysaccharides.

[37] Chen X., Wu J.H., Li X.Z., Yang F.J., Yu L.H., Li X.K., Wang S.Y. (2022). Investigation of the Cryoprotective Mechanism and Effect on Quality Characteristics of Surimi During Freezing Storage by Antifreeze Peptides. Food Chem..

[38] Matthew A.N., Horst K., Clifford L.W. (2014). The Effect of Free Ammonia Nitrogen, pH and Supplementation with Oxygen on the Growth of South African Abalone, Haliotis midae L. in an Abalone Serial-use Raceway with Three Passes. Aquacult. Res..

[39] Tian H.M., Zhang Y.F., Yang X., Chen L.Q., Qin X.M., Wang K.S., Zhu Q.J. (2025). Metal-polyphenol Network Nanocomposites Loaded Degradable Films with “On-off” Antibacterial Ability for Cooked Meat Storage. Chem. Eng. J..

[40] Tan H., Wu B., Li C.P., Mu C.D., Li H.L., Lin W. (2015). Collagen Cryogel Cross-linked by Naturally Derived Dialdehyde Carboxymethyl Cellulose. Carbohydr. Polym..

[41] Zhang K., Hou H., Bu L., Li B.F., Xue C.H., Peng Z., Su S.W. (2017). Effects of Heat Treatment on the Gel Properties of the Body Wall of *Sea cucumber* (*Apostichopus japonicus*). J. Food Sci. Technol..

[42] Zhang C.P., Qu G.W., Wang Q.T., Zhao Y.P., Xie Y.L., Xu S.M., Sun L.L. (2024). Comparative Effects of in-package High Pressure Steam Sterilization and High Temperature Boiling on the Quality Changes and Shelf Life of Ready-to-eat *Sea cucumber*. J. Food Compos. Anal..

[43] Lund M.N., Heinonen M., Baron C.P., Estévez M. (2011). Protein Oxidation in Muscle Foods: A Review. Mol. Nutr. Food Res..

[44] Moradi M., Divsalar A., Saboury A.A., Ghalandari B., Harifi A.R. (2015). Inhibitory Effects of Deferasirox on the Structure and Function of Bovine Liver Catalase: A Spectroscopic and Theoretical Study. J. Biomol. Struct. Dyn..

[45] Bi C.H., Chi S.Y., Zhou T., Zhang J.Y., Wang X.Y., Li J., Liu Y. (2022). Effect of Low-frequency High-Intensity Ultrasound (HIU) on the Physicochemical Properties of Chickpea Protein. Food Res. Int..

[46] Kamińska A., Sionkowska A. (1996). Effect of UV radiation on the infrared spectra of collagen. Polym. Degrad. Stabil..

[47] Liu J.Y., Chen S.Y., Jiang X.M., Xue C.H., Cao H.H., Liu D.P., Sun X. (2021). Microbial Transglutaminase Inhibits the Deterioration of High-temperature-treated *Sea cucumber*. J. Food Process. Preserv..

[48] Wang J., Lin L., Sun X., Hou H. (2020). Mechanism of *Sea cucumbers* (*Apostichopus japonicus*) Body Wall Changes Under Different Thermal Treatment at Micro-scale. LWT.

[49] Song Y.Q., Yao G.L., Chen J., Li N. (2023). Effect of β-cyclodextrin on whey protein-epigallocatechin gallate interaction. Ind. Crops Prod..

[50] Zhong Y., Khan M.A., Shahidi F. (2007). Compositional Characteristics and Antioxidant Properties of Fresh and Processed *Sea cucumber* (*Cucumaria frondosa*). J. Agric. Food Chem..

[51] Sasimontra T., Kowit H., Janjira I., Kornkanok I., Yasuteru S., Nutchaninad T., Prapapan T. (2025). Comprehensive Evaluation of Tectona grandis L.f.: Integrated In Vitro and In Silico Analysis of Steroid 5α-Reductase Inhibition and Anti-Inflammatory Properties. ACS Omega.

